# Acute pancreatitis induced by valproic acid

**DOI:** 10.1192/j.eurpsy.2021.1706

**Published:** 2021-08-13

**Authors:** S. Khouadja, R. Melki, C. Ben Taleb, L. Zarrouk

**Affiliations:** Psychiatry, University Hospital Of Mahdia, Mahdia, Tunisia

**Keywords:** pancreatitis, psychiatry, bipolar disorder, valproic acid

## Abstract

**Introduction:**

Valproic acid (VPA) is a commonly prescribed medication for epilepsy, migraine and especially bipolar disorder therapy. Although the common adverse effect associated with VPA are typically benign, less common adverse effects can occur; these include acute pancreatitis.

**Objectives:**

Describe the clinical and therapeutic characteristics of a case of acute pancreatitis induced by VPA with a review of the literature.

**Methods:**

We report the case of a patient who presented an acute pancreatitis induced by VPA. The data was collected from the patient’s medical file. A review of the literature was performed by selecting articles from the PubMed search engine using ‘acute pancreatitis and valproic acid’ and ‘drug induced acute pancreatitis’ as key words.

**Results:**

This is a 51-year-old male patient with a history of type 2 diabetes, dyslipidaemia and psychiatric follow-up for bipolar disorder type I on lithium. He was admitted for a resistant depressive episode. We opted for the combination of two mood stabilizers (VPA and lithium). On the third day of treatment, the patient reported epigastric pain with incoercible vomiting. Laboratory tests showed increased levels of pancreatic enzymes and a biological inflammatory syndrome. The diagnosis of acute stage A pancreatitis was made.VPA was discontinued and the patient was put on symptomatic treatment with favourable outcome after one week. The etiological investigation ruled out other causes of acute pancreatitis. As a result, iatrogenic origin was retained.

**Conclusions:**

This case supports the idea that acute pancreatitis may be induced by VPA, it has no predictable factors.

**Disclosure:**

No significant relationships.

